# Rival assessment among northern elephant seals: evidence of associative learning during male–male contests

**DOI:** 10.1098/rsos.150228

**Published:** 2015-08-12

**Authors:** Caroline Casey, Isabelle Charrier, Nicolas Mathevon, Colleen Reichmuth

**Affiliations:** 1 Department of Ecology and Evolutionary Biology, and, University of California Santa Cruz, Santa Cruz, CA 95060, USA; 2 Institute of Marine Sciences, Long Marine Laboratory, University of California Santa Cruz, Santa Cruz, CA 95060, USA; 3 Equipe Communications Acoustiques, Neuro-PSI, CNRS UMR 9197, Université Paris Sud, 91405 Orsay, France; 4 Equipe Neuro-Ethologie Sensorielle, ENES/Neuro-PSI, CNRS UMR 9197, Université de Lyon/Saint-Etienne, 23 rue Michelon, 42023 Saint-Etienne cedex 2, France

**Keywords:** male–male conflict, acoustic communication, playback experiments, social network, individual recognition, *Mirounga*

## Abstract

Specialized signals emitted by competing males often convey honest information about fighting ability. It is generally believed that receivers use these signals to directly assess their opponents. Here, we demonstrate an alternative communication strategy used by males in a breeding system where the costs of conflict are extreme. We evaluated the acoustic displays of breeding male northern elephant seals (*Mirounga angustirostris*), and found that social knowledge gained through prior experience with signallers was sufficient to maintain structured dominance relationships. Using sound analysis and playback experiments with both natural and modified signals, we determined that males do not rely on encoded information about size or dominance status, but rather learn to recognize individual acoustic signatures produced by their rivals. Further, we show that behavioural responses to competitors' calls are modulated by relative position in the hierarchy: the highest ranking (alpha) males defend their harems from all opponents, whereas mid-ranking (beta) males respond differentially to familiar challengers based on the outcome of previous competitive interactions. Our findings demonstrate that social knowledge of rivals alone can regulate dominance relationships among competing males within large, spatially dynamic social groups, and illustrate the importance of combining descriptive and experimental methods when deciphering the biological relevance of animal signals.

## 1. Introduction

Theoretical models of animal conflict predict it is advantageous for males to accurately assess rivals when competing for females [1,2]. Consequently, signals that encode relevant information about the fighting ability of senders help receivers to determine appropriate behavioural responses, and thus reduce the costs of conflict in terms of energy expenditure, injury or even death [1,3]. Rival assessment is often based on signal features that correspond to resource-holding potential [4–6] or motivational state [7]. Alternatively, an individual may remember the outcome of previous competitive interactions with an opponent, and learn to associate these consequences with a signal emitted by the individual with whom he previously fought. This information can then be used to influence decision-making during later encounters. The latter situation has been described for species living in small stable social groups, where mechanisms for individual recognition allow for the formation of linear hierarchies based on frequent interactions between group members [8–10]. It is unclear whether similar associative learning processes—based on individual recognition—can support structured dominance hierarchies within very large and fluid social groups. However, we can hypothesize that remembering one's previous opponent could be the most secure strategy for rival assessment when both the competition level and the cost of physical fights are extremely high. In a system where great size and strength are traits of any male who survives to adulthood, signals conveying honest information about male quality may not be that informative, and other means for rival assessment may be present.

Owing to extreme selection pressures for rival assessment, the northern elephant seal (*Mirounga angustirostris*) provides an optimal social model to explore how signals can be used to mediate competitive behaviour among breeding males. Reproduction in this species is annually synchronous, and mature females congregate by the hundreds or thousands on beaches to give birth and breed [11]. Adult males arrive at breeding sites before the females and remain ashore until after the females have departed—a tenure that may span 100 days without access to food or water [11]. Compared to females, males live markedly shorter lives [11]: only 5% survive to physical maturity [12] with less than 1% gaining reproductive access to females [11]. This asymmetry in life history and reproductive success underpins one of the most competitive breeding systems known among mammals.

Male northern elephant seals fiercely compete to control access to female harems during the breeding season. While social status is initially established through physical confrontations [13], dominance relationships between familiar individuals are maintained by ritualized displays that include loud vocalizations, elevated visual posturing and seismic cues produced by slamming the chest against the substrate [14,15]. The directed displays emitted by higher ranking males are usually sufficient to control the movements of subordinates relative to female harems. Thus, while behavioural exchanges between competing males are common, physical battles are relatively rare [14] and extremely costly [16].

The vocalizations produced by males during their displays, traditionally called ‘clap threats’, contain 3–20 broadband units emitted at high levels with repetition rates of a few pulses per second [17]. These signals appear to efficiently transmit information about the level of threat presented by the caller, even in situations where visual cues are unavailable [18]. Vocal playbacks have been shown to elicit movement from other males on the rookery [19] with individual responses to playbacks influenced by both caller orientation [20] and relative social status [21]. Early investigators commented on apparent individual differences in the calls of competing males, and indicated that these acoustic differences may be attributable to differences in the size and/or status of callers [22]. Subsequent behavioural and acoustic analyses confirmed the presence of reliable individual differences [15,23], but provided no indication that a male's call structure is associated with his dominance status [23]. These findings suggested that male calls could function to convey individual identity, and that individuals may learn to associate distinctive features of a threat call with a specific male through learned association [15,23]. At present, it remains unknown whether the acoustic displays of male northern elephant seals function as honest signals that opponents can decode without prior experience, or whether they are individual identifiers which males must learn in order to economize their effort during the energetically demanding breeding season.

To investigate the information contained in the signals emitted by adult male northern elephant seals, we performed a multi-year study that integrated information about the morphological features, spatial relationships and competitive interactions of known individuals with fine-scale acoustic analyses of their vocal displays. We then applied these results to field playback experiments that explored the extent to which receivers actually used information encoded in vocalizations during conflicts. Specifically, we experimentally tested three alternative hypotheses:

(i) calls encode resource-holding potential, and there are correlations between the acoustic features of an individual's call and his morphological traits and/or dominance rank. Males should thus use these acoustic features to modulate their responses to competitors;
(ii) males cannot or do not depend on vocal features signalling phenotype and/or dominance. Individuals then should learn how to respond to rivals from experience only, and a reliable individual vocal signature should support this process; or
(iii) there is a mixed system in which males respond differently to the calls of familiar versus unfamiliar individuals. In this case, males may depend on acoustic cues linked to resource-holding potential to modulate their responses to unknown competitors, while relying on individual vocal signature and previous experience when responding to familiar rivals.

The results of this work provide insight into the function of this specialized acoustic signal and demonstrate its role in maintaining dominance relationships within relatively large social networks that are not spatially predicable. Our findings also shed light on the outstanding question of behavioural flexibility in decision-making during male–male contests.

## 2. Material and methods

### 2.1 Study animals

**Figure 1.**
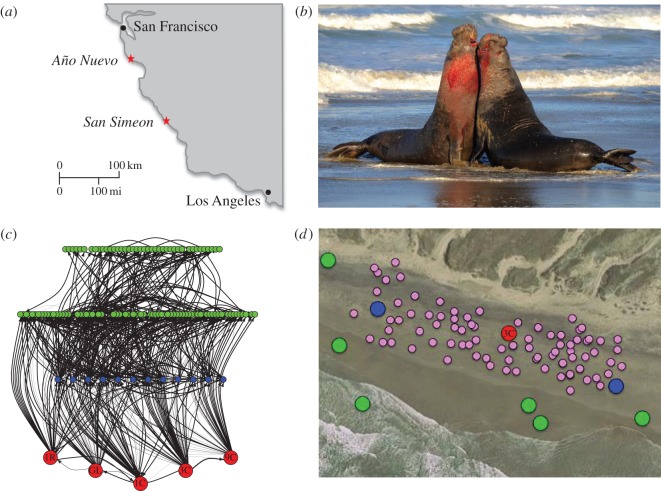
(*a*) Locations of the primary (Año Nuevo) and remote (San Simeon) study sites. (*b*) Example of male–male physical conflict following exchange of displays. (*c*) The social network of northern elephant seal males as it appeared at the Año Nuevo study site during the 2010–2011 reproductive season. Red dots=‘alpha’ males that controlled female harems over the season; blue dots=‘*beta*’ males that reliably held harem flanking positions with opportunistic access to females; green dots=‘peripheral’ males that typically lacked access to harems (the ‘peripheral’ males shown on the upper layer of the network were never observed interacting with alphas). Each arrow represents a directed interaction, drawn from a winner to a loser. The thickness of the arrows is proportional to the number of observed competitive events between two males (mean±s.d.=1.95±2.02 events; range 1–19; 1170 competitive events; see Methods for details on data collection). Each alpha male interacted with 37.6±3.7 individuals (range 33–43). The number of interactants for beta males was 26.1±10.5 (range 10–39). More than 97% of relationships between males were fully asymmetric (i.e. the interactions within a given dyad of males were always won by the same individual). The dyad is thus characterized by a well-established ‘dominant–subordinate’ relationship, illustrating the stability of the hierarchy between males along the reproductive season. (*d*) Aerial schematic of a harem. Red, blue, and green dots represent ‘alpha’, ‘beta’ and ‘peripheral’ males respectively; pink dots represent females.

We worked at Año Nuevo State Park (37.1086 N, −122.338 W) from December through to February for four consecutive years from 2009 to 2013, and 300 km south at San Simeon State Park (35.6512 N, −121.2196 W) during the 2011–2012 breeding season (figure 1*a*). At Año Nuevo, we dye-marked 30–51 adult males (aged 8–14 years) annually upon their first sighting in our study area—a 1 km long section of sandy beach used by approximately 300 adult females. A subset of adult males also had flipper tags for inter-annual identification. We took calibrated photographs of the males and recorded their location each observation day to determine proximity to female harems as well as to assess site fidelity, movement patterns and rival familiarity. Fewer than 20 adult males were reliably re-sighted at the Año Nuevo study area during each breeding season. At the San Simeon site, we marked and photographed 15 adult males.

### 2.2 Determining the dominance status of males

To evaluate the dominance status of each individual, determine the relative size of his social network and quantify the use of vocalizations during competitive behaviour, at least two experienced observers scored dyadic interactions between identified males throughout each breeding season. For each interaction, we recorded the identity of the apparent winner, whether he had vocalized and how far he had moved. The same information was recorded for the apparent loser. We also recorded whether there was physical contact between the two males (figure 1*b*), and if so, scored the intensity of the interaction (from single blows to sustained combat). The dominance status of males, including those that had not directly interacted with one another, was then determined by applying an Elo-rating approach to these data, which assigns a quantitative score to individuals based on the probability of one individual beating another in a two-player game [24–26]. Each male was assigned an initial Elo score of 1000, as every individual began the season with the same presumed probability of winning a dyadic competitive interaction. We then adjusted individual Elo scores after each observed interaction by an amount proportional to the expected outcome, such that their subsequent win/lose probabilities changed with their adjusted rating. Relative probability (*E*_*a*_) that individual 1 would beat individual 2 was determined using the equation $E_{a}=1/1+10^{\left({\mathrm{Rank}}_{2}-{\mathrm{Rank}}_{1}/400\right)}$, where Rank_2_ and Rank_1_ are the ranks of individuals 2 and 1 prior to the observed interaction. Ratings were modified in proportion to the deviation from the anticipated outcome, and new Elo scores were determined using the formula $R_{A}^{\prime }=R_{A}+K(S_{A}-E_{a})$, where $R_{A}^{\prime }=\text{new rank}$, *R*_*A*_=previous rank, *K*=constant, *S*_*A*_=actual score (1 for winner, 0 for loser) and *E*_*a*_ is the probability of each individual winning that interaction. Elo calculations were executed in R (custom code developed in R Development Core Team 2004, www.R-project.org).

Elo scores provided an instantaneous measure of dominance as well as an overall (seasonal) dominance score for each individual. At the end of each breeding season, the final Elo score for each male in the sample was validated against his corresponding descriptive rank, which in turn was qualitatively based on his repeatedly sampled proximities to female harems (§2.1). Alpha males held stable positions within female harems, beta males held flanking positions relative to harems, and peripheral males were totally excluded from access to harems (figure 1*c*,*d*). Elo scores were calculated for focal males both at the Año Nuevo and San Simeon field sites.

### 2.3 Assessment of morphological traits and age

**Figure 2.**
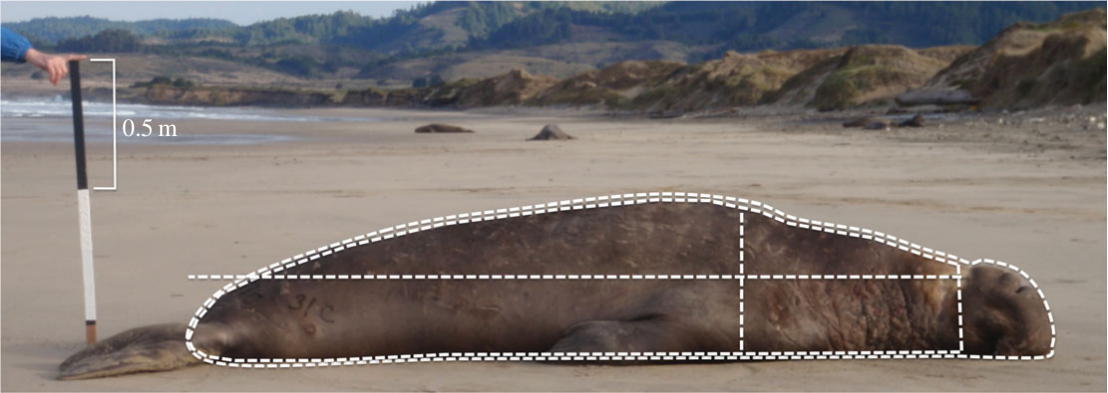
Assessment of morphological features. Photographs were taken with a calibrated scale to determine four parameters of body size: length (from front of eye to base of tail), vertical height (at axilla), total body perimeter and head perimeter.

To estimate the size of focal males, we analysed digital photographs obtained with a scale bar positioned on axis with the midline of the animal. The photographs were taken while individuals were lying at rest with straight (supine) body posture on flat terrain. The images were analysed with ImageJ (v. 1.34, National Institutes of Health) to determine four parameters of body size: length, vertical height, body perimeter and head perimeter (figure 2). The age class of focal males was determined in the field based on scarring of the chest shield, development of the proboscis and body length (as in [27,28]); age class estimates were later verified from scaled photographs by experienced, independent observers.

### 2.4 Recording calls

To provide a large dataset of male vocalizations for acoustic analysis, we opportunistically recorded males from 5 to 15 m during stereotyped displays using a Neumann KMR 82i Condenser Shotgun Microphone (with Rycote suspension and windscreen) connected to a Fostex FR-2 Field Memory Recorder (24-bit/48 kHz). Additionally, to determine call source levels, we used a calibrated Brüel and Kjær 4189 condenser microphone (with UA-1650 windscreen) held at 1 m from, and on-axis with, the head of the animal. Signals were received by a Brüel and Kjær 2250 sound-level metre (24-bit/48 kHz). Recordings were obtained throughout the breeding season. To determine the stability of an individual's call across different behavioural states, the social context of every recorded vocalization was categorized as either directed (emitted towards another male) or non-directed (produced when not interacting with another individual). It was possible to record vocalizations at close ranges without disturbance to male seals, as adults typically completed each acoustic display once initiated, regardless of external cues or the presence of researchers.

### 2.5 Acoustic analysis of calls

#### 2.5.1 Measurements

To examine individual variation in call structure, we characterized the calls of focal adult males (more than 8 years old) using the acoustic recordings obtained during competitive interactions. These males held mid-to-high ranks in the dominance hierarchy, including alpha, beta and peripheral positions (figure 1*c*,*d*). All recorded calls were evaluated and subjectively scored for quality. Only calls with low background noise and without overlapping acoustic signals were used in subsequent analyses. To avoid possible replication of individuals in the study over multiple years, we used only calls from identified individuals recorded within a single season (2010–2011) for this analysis.

**Figure 3.**
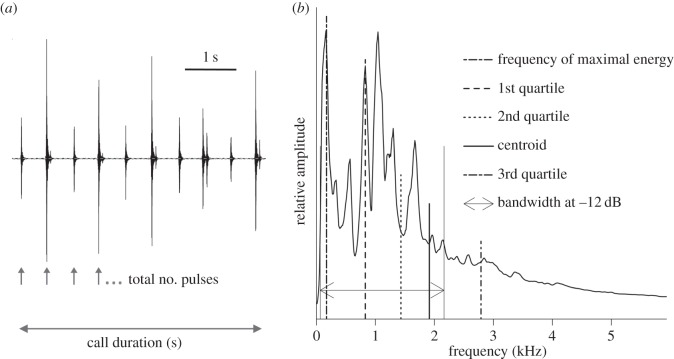
Measurement of temporal and spectral acoustic parameters. (*a*) Sample call waveform. We measured the total number of pulses over the call, the call duration and calculated the number of pulses per second to get the pulse rate. (*b*) Call frequency spectrum. To characterize the call frequency content, we measured the frequency of maximal energy, the frequencies at each quartile of total energy (Q25 at 25%, Q50 at 50% and Q75 at 75%), the spectrum centroid and the −12 dB bandwidth.

Based on the quantity and quality of the available recordings, we described the calls of 16 individuals in both the temporal and spectral domains (mean: 15.8 calls individual^−1^, range: 9–25 calls individual^−1^). We selected acoustic parameters that could be applied to all calls. To assess temporal features, we used Avisoft SAS Lab Pro to perform a pulse train analysis on the normalized envelope of the main (rhythmic) portion of the call, excluding introductory and terminal snorts (smooth: 41 pts, frequency range 0–6 kHz), and measured the following parameters: call duration (s), total number of pulses (*n*) and the average repetition rate (pulse rate, Hz; figure 3*a*). The transient and broadband structure of the entire call precluded a traditional analysis of energy distribution among frequencies. Several spectral features were measured over the same portion of the call with the Seewave R package [29]: the centroid of the frequency spectrum (Hz), the 25%, 50% and 75% frequency quartiles (1st quartile: ‘Q25’, 2nd quartile: ‘Q50’ and 3rd quartile: ‘Q75’, in Hz), the frequency bandwidth within which the energy falls within 12 dB of the maximal frequency peak (Hz), and the frequency of maximal energy (Hz; figure 3*b*). Individuals showed reliable substructure within the repeatable units comprising the rhythmic portion of each call; these patterns were identified and descriptively coded but not included in subsequent analyses. In contrast to analyses of the calls of the congeneric southern elephant seal (*Mirounga leonina*) [30], we did not assess frequency information such as *F*_0_ or formant structure in this study—the calls of northern elephant seals are discrete pulses rather than long roars, and therefore preclude such measures.

Fifteen individuals from the same season had at least four high-quality source level recordings and measurements. Given the impulsive nature of these calls, amplitude was reported as dB_peak_ at 1 m (referenced to 20 μPa) rather than as dB_RMS_ sound pressure level.

#### 2.5.2 Individual signatures

To determine whether there were reliable differences among the calls of individuals, we used a cross-validated and permuted discriminant function analysis (pDFA [31,32]; customized script written in R). A fitting dataset (two-third of the calls from each individual) was used to generate linear discriminant functions on the basis of the acoustic features describing the calls. The remaining one-third of the calls were used as a cross-validation set to measure the percentage of correctly classified vocalizations. The mean effect size was calculated from 100 random iterations. From the cross-validation results, we extracted a confusion matrix to show the conditional probability that a call emitted by the individual *i* was in fact emitted by *j*: confusion(*i*,*j*)=*p*(*i*|*j*). To determine the significance of the effect size calculated by the cross-validation step, we created datasets where the identity of calls was randomly permuted between individuals (pDFA). For each of these randomized sets, we followed the same steps—training and validation—as with the non-randomized sets. After 1000 such iterations, we calculated the proportion of randomized validation datasets with the number of correctly classified calls being at least as large as the effect size obtained with the non-randomized validation dataset. This proportion gives the significance of the level of discrimination and is equivalent to a *p*-value [33].

#### 2.5.3 Variability over years and across social contexts

To assess the long-term reliability of call structure, we recorded a subset of individuals over two successive years (*n*=10 males, 5.8 calls individual^−1^ year^−1^, range: 5–6), and measured both the centroid of the frequency spectrum and the pulse rate (main parameters shown to support the individual signature—see Results). We then calculated Euclidian distances in the two-dimensional space defined by these two parameters after transforming them into *Z*-scores. Three categories of distances were computed: within the calls recorded during year 1 for each individual, between the calls of years 1 and 2 for each individual, and between the calls of year 1 of each individual and all the other calls of all other individuals from year 1. We then evaluated whether the average distance between an individual's calls during year 1 was shorter than the average distance between its year 1 and year 2 calls. We also calculated densities of the distribution of the three categories of Euclidian distances. We followed a similar procedure to assess the stability of vocal signatures between calls produced in directed versus non-directed social contexts (*n*=8 males, 4.7 calls individual^−1^ social context^−1^, range: 2–6).

### 2.6 Correlations between acoustic cues, body size and dominance

To investigate whether spectral, temporal and/or acoustic features were linked to morphological traits or dominance score among the focal males, we performed linear correlations (lm function in R statistical package). We also assessed whether the morphological traits of the focal males was correlated with their dominance scores.

### 2.7 Playback experiments

#### 2.7.1 General procedure

Playback tests were performed during periods of high male responsiveness corresponding to the females' oestrous. Adult males were tested once or twice a day, with at least 3 h separating each test to avoid habituation. We used a similar method to that described by Holt *et al*. [20]. Playback signals were projected from a self-powered Premio 8 PA sound system or paired Advent AV570 speakers capable of replicating the amplitude and spectral components of the recorded calls. The speaker was placed 7±1 m from the focal male, except as noted in §2.7.3 (experiments on alpha males). To control for possible directionality effects, the speaker was placed on axis with the focal animal (maximum deviation 90^°^). Males were challenged after a minimum period of 2 min of not having interacted with other males, and with no other males within a 7 m radius. Each playback included three different calls separated by 3 s of silence and broadcast at 116±1.5 dB_peak_ at 1 m. For playback experiments using modified calls (described in §2.7.2), we built each series with only two repetitions of a single call; this was done to limit habituation since each of the 10 adult males was tested with up to seven different signals.

#### 2.7.2 Testing the use of size-related acoustic cues

To evaluate the biological relevance of acoustic features that scaled with body size, we challenged 10 adult males from Año Nuevo, ranging in eye-to-tail length from 3.2 to 3.6 m, with playbacks of signals derived from those recorded in a distant colony (Piedras Blancas, San Simeon State Park, CA, USA) to avoid familiarity with senders. We mimicked either smaller males (less than 3.2 m) or larger males (more than 3.6 m) by changing the characteristics of natural calls that were shown to be correlated with size: pulse rate, the number of pulses or the frequency spectrum (see Results). Temporal modifications of natural calls were made by deleting or adding pulses to alter the pulse number, and by shortening or lengthening the inter-pulse interval to alter the pulse rate. Spectral content (modified Q25) was manipulated by re-synthesizing natural calls using a PSOLA-based algorithm in PRAAT [33].

In the first set of experiments, we played back calls with modified pulse rates (1, 1.7 or 3 Hz, corresponding to small, medium and large males, respectively), while the number of pulses per call remained fixed (14 pulses, a rate corresponding to average-sized males). In the second set of experiments, we modified the number of pulses per call (7, 14 or 21 pulses, corresponding to small, medium and large males, respectively) while maintaining an average pulse rate (1.7 Hz). Finally, we challenged the same males with signals showing both fixed pulse rate (1.7 Hz) and number of pulses (14) but with modified spectral content (either a low Q25 of 536 Hz or a high Q25 of 804 Hz; these low and high Q25 values correspond to mean Q25 values approximately ±20%, representing large and small males, respectively). These experiments were conducted during the 2011–2012 breeding season.

#### 2.7.3 Testing the effect of social rank and familiarity

To assess whether alpha and beta males have the same responsiveness to the dominance status and/or the familiarity of the callers, we performed three sets of playback experiments. First, we tested whether beta males respond differently to calls from known dominant and subordinate males. We challenged 10 beta-ranking males at Año Nuevo with calls from both dominant and subordinate familiar rivals. Target males were sighted for at least 10 days before the experiments and their social ranks determined (harem flanking males with Elo scores of 964–1713). The playback treatments for each target male were selected based on at least three observed interactions in which the familiar rival had called, and there was a clear approach or retreat response by the target male. These experiments were conducted during the 2010–2011 breeding season.

In a subsequent experiment, we tested whether the observed responses of males to the calls of familiar rivals were dependent on prior experience with an individual. In this test, we used the calls from the same dominant–subordinate playback treatments to challenge 10 different beta males from a distant colony (San Simeon). We took great care to match the dominance status of these naive males with the 10 beta males tested previously. These experiments were conducted during the 2011–2012 breeding season.

Finally, owing to their high dominance status on the rookery, alpha males could not be tested with both familiar dominant and subordinate treatments. Rather, to evaluate their responsiveness to imposing males on the rookery, five alpha males from Año Nuevo (harem-holding males with Elo scores of 1056–2047) were challenged with calls from neighbouring alphas, familiar (flanking) betas and unfamiliar alpha males (males recorded the same year but in another area of the breeding colony and never seen at our study site). We performed these playbacks at four successive distances along a linear transect from the border of the alpha's harem (40, 30, 20 and 10 m) to simulate intrusion of an approaching adult male. These experiments were conducted during the 2010–2011 breeding season.

### 2.8 Analysis of responses to playbacks

The behavioural responses of target males to playbacks were measured over a 90 s period from the onset of the playback, and characterized by six measures: latency to orient towards the loudspeaker (s), latency to change posture (s), latency to vocalize (s), number of emitted calls, latency to move towards or away from the loudspeaker (s) and distance moved (m). Rather than separately analysing these six non-independent measures of response, they were collapsed using a principal component analysis (PCA, varimax rotations [6,34,35]. The PC scores of components showing eigenvalues of more than 1 were used to compare responses to different stimuli. For playbacks using modified calls (§2.7.2) and those with dominant/subordinate pairs (§2.7.3), we used non-parametric tests (Wilcoxon matched pairs tests [36]). To cope with the 2×2 fully crossed design (playback type×distance) of the test on alpha males, we used a linear mixed model (function lmer in R lme4 package), after transforming data to meet the model assumption (exponential transformation), and checking the distribution of the residuals with respect to normality and homoscedasticity (fixed effects: playback type and distance; random effects: intercepts for tested males, males random slopes for the effect of playback type and distance [36]). *p*-values were obtained with likelihood-ratio tests comparing the fit of the full model with reduced models lacking playback type or distance.

## 3. Results

### 3.1 Social interactions and the use of vocalizations

We observed and scored 2445 male–male dyadic competitive interactions over four breeding seasons (table 1). Most interactions involved approach and/or multi-modal display behaviour after which one individual assumed a submissive posture and retreated (electronic supplementary material, video S1). Vocal displays from at least one individual of the pair were observed in 76% of interactions. Winners called during 95% of interactions that included vocalizations, much more frequently than losers (29%). Only 5% of interactions led to physical contact. Sustained fights comprised less than 2% of the interactions, and occurred most often when neither male backed down from an escalating dispute. The majority of these battles involved vocalizations from both individuals and all occurred between males of similar dominance status that had not fought previously that season. Within a given season, alpha and beta male elephant seals engaged competitively with an average of 38 and 26 other males, respectively (figure 1*c*).

**Table 1.** Summary of observed frequency of events within and across breeding seasons, including the relative proportions (%, by type) of male competitive interactions. (Unique males are those that have been marked at the primary field site during a given season, a subset of these are present across two or more seasons. Consistent scoring of vocal behaviour throughout each breeding season indicated that the observed trends in vocal signalling by winners and losers during competitive interactions were similar year- to-year.)

breeding season event	2010	2011	2012	2013	total
days with observations	23	49	31	24	127
hours of opportunistic sampling	43	191	82	90	406
number of calls recorded	800	1468	584	759	3611
unique males identified	74	177	79	142	320
unique adult males identified	30	50	40	51	171
unique males observed interacting	48	127	61	116	352
interactions observed	383	1177	786	482	2445
unique males with more than five interactions	24	64	36	30	130
unique males with more than 10 interactions	16	42	33	26	101
winner vocalized interactions	—	863	590	334	1787
% winner vocalized interactions		73.3	75.8	69.3	73.4
loser vocalized interactions	—	255	186	105	546
% loser vocalized interactions		21.8	23.7	21.9	22.4
neither vocalized interactions	—	258	179	136	573
% neither vocalized interactions		22.1	22.8	28.3	23.5
both vocalized interactions	—	242	175	105	522
% both vocalized interactions		21.5	22.5	21.8	21.4
physical contact interactions	—	65	35	24	124
% physical contact interactions		5.6	4.5	5.0	5.1
sustained physical contact interactions	—	21	11	12	44
% sustained physical contact interactions		1.8	1.4	2.5	1.8
sustained physical contact interactions—both vocalized	—	10	6	8	24
% sustained physical contact interactions—both vocalized		47.6	54.5	66.7	54.5
sustained physical contact ends without decision	—	7	2	0	9
% fights end as draw		33.3	18.2	0.0	20.5

### 3.2 Relationships between acoustic cues, body size and dominance

Call pulse rate and the total number of pulses per call were positively correlated with most body measurements (table 2). Further, the first frequency quartile (Q25) decreased with body size, with larger animals having lower frequency calls (table 2). None of the acoustic parameters were correlated with dominance score among breeding age males (table 2). There was also no correlation between dominance score and morphological traits among adult males (table 3).

**Table 2.** Correlation of acoustic parameters with morphological measures and dominance rating. (Note that significant correlations are shown in bold.)

acoustic parameter	*n*	*R*^2^ (adjusted value)	equation	*p*-value
correlations with vertical height				
duration (s)	16	−0.057	4.72*x*+6.10	0.665
**pulse rate (Hz)**	**16**	**0**.**425**	**3**.**11*x***−**0**.**68**	**0**.**004**
number of pulses	16	0.138	29.6*x*−6.47	0.086
***F***_**max**_**(Hz)**	**16**	**0**.**282**	**676*x*-152**	**0**.**020**
centroid (Hz)	16	0.059	575*x*+1294	0.185
**Q25 (Hz)**	**16**	**0**.**413**	**1693*x***−**642**	**0**.**004**
Q50 (Hz)	16	0.040	695*x*+699	0.221
Q75 (Hz)	16	0.002	731*x*+1877	0.329
−12 dB bandwidth (Hz)	16	−0.070	0.124*x*+1.0	0.891
dB_peak_ re: 20 μPa at 1 m	15	−0.027	−4.99*x*+130	0.425
correlations with body length				
duration (s)	16	0.090	5.01*x*−7.14	0.137
**pulse rate (Hz)**	**16**	**0**.**489**	**1.11*x***−**2.26**	**0**.**002**
**number of pulses**	**16**	**0**.**263**	**12.8*x***−**28.5**	**0**.**024**
*F*_max_ (Hz)	16	0.171	152*x*−221	0.062
centroid (Hz)	16	−0.003	118*x*+1294	0.346
**Q25 (Hz)**	**16**	**0**.**262**	−**169*x***−**1233**	**0**.**033**
Q50 (Hz)	16	0.041	197*x*+517	0.221
Q75 (Hz)	16	−0.036	143*x*+1903	0.500
−12 dB bandwidth (Hz)	16	−0.069	0.057*x*+0.906	0.861
dB_peak_ re: 20 μPa at 1 m	15	−0.129	−2.76*x*+5.49	0.123
correlations with body perimeter				
duration (s)	16	−0.021	1.57*x*−2.26	0.420
**pulse rate (Hz)**	**16**	**0**.**454**	**0.677*x***−**3.55**	**0**.**003**
**number of pulses**	**16**	**0**.**250**	**6.44*x***−**33.7**	**0**.**028**
*F*_max_ (Hz)	16	0.187	120*x*−599	0.053
centroid (Hz)	16	−0.022	63.6*x*+1219	0.426
Q25 (Hz)	16	0.161	−73.2*x*+1205	0.138
Q50 (Hz)	16	−0.007	90*x*+508	0.359
Q75 (Hz)	16	−0.049	74*x*+1832	0.591
−12 dB bandwidth (Hz)	16	−0.062	−0.05*x*+1.46	0.731
dB_peak_ re: 20 μPa at 1 m	15	0.162	−1.91*x*+141	0.096
correlations with head perimeter				
duration (s)	16	−0.063	−2.44*x*+10.6	0.737
pulse rate (Hz)	16	0.106	2.13*x*+0.20	0.117
number of pulses	16	−0.070	−2*x*+13.45	0.884
*F*_max_ (Hz)	16	−0.056	290*x*+81.1	0.662
centroid (Hz)	16	−0.052	152*x*+1607	0.621
Q25 (Hz)	16	−0.069	−129*x*+731	0.601
Q50 (Hz)	16	−0.066	101*x*+1121	0.787
Q75 (Hz)	16	−0.034	350*x*+2188	0.488
−12 dB bandwidth (Hz)	16	−0.071	0.011*x*+1.08	0.990
dB_peak_ re: 20 μPa at 1 m	15	−0.081	1.11*x*+126	0.755
correlations with dominance rating				
duration (s)	16	0.053	−0.0044*x*+15.2	0.197
pulse rate (Hz)	16	0.051	−0.0005*x*+2.03	0.201
number of pulses	16	−0.04	−0.0031*x*+16.7	0.528
*F*_max_ (Hz)	16	−0.070	−0.0098*x*+257	0.940
centroid (Hz)	16	−0.070	−0.004*x*+1700	0.970
Q25 (Hz)	16	−0.050	0.1*x*+532	0.144
Q50 (Hz)	16	−0.060	0.056*x*+1103	0.704
Q75 (Hz)	16	−0.065	−0.05*x*+2452	0.777
−12 dB bandwidth (Hz)	16	−0.069	0.00006*x*+1.01	0.852
dB_peak_ re: 20 μPa at 1 m	15	−0.081	1.11*x*+126	0.755

**Table 3.** Correlation of dominance status (Elo score) with morphological measures.

	*n*	*R*^2^ (adjusted value)	equation	*p*-value
vertical height	16	0.038	−1279*x*+2300	0.228
body size	16	−0.015	−311*x*+2463	0.391
body perimeter	16	−0.0008	−199*x*+2900	0.337
head perimeter	16	−0.068	146*x*+1342	0.839

### 3.3 Size information and its influence on rivals' behaviour

**Figure 4.**
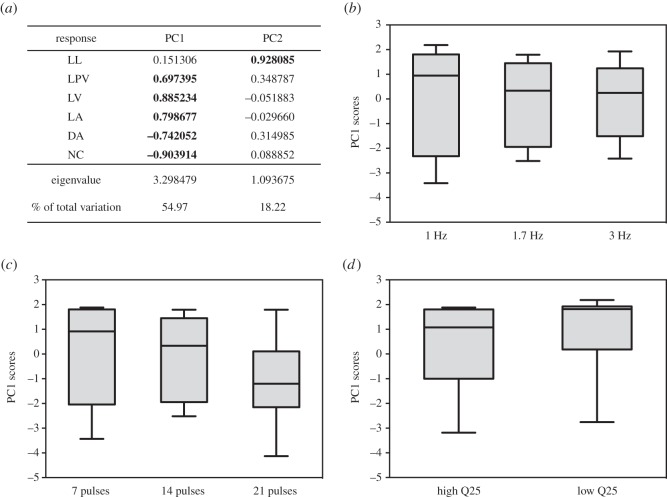
Behavioural responses to modified calls. (*a*) Results of the PCA performed on the different behavioural measurements (latencies to look LL, for posture change LPC, to vocalize LV and to approach LA; distance of approach DA; number of calls NC). Bold values indicate behavioural variables that were strongly correlated to PC scores. (*b*) Responses to signals with modified pulse rate. (*c*) Responses to signals with different number of pulses. (*d*) Responses to lower and higher pitched calls. None of these signal variations produced significant differential responses in the males that were tested (*n*=10). The whiskers on box plots show min-max.

We determined whether focal males used the morphological information encoded within vocalizations by modifying the body-size-linked acoustic features of call (pulse rate, total number of pulses per call and first frequency quartile) during playback experiments. Only the first two components of the PCA (PC1 and PC2) performed on the six behavioural measurements showed eigenvalues of more than 1, and explained 55% and 18% of the total variance, respectively (figure 4). All the behavioural variables except latency to orient were strongly correlated to PC1, with distance moved and number of calls negatively correlated to PC1 (factor loadings: latency to orient: 0.151; latency to change posture: 0.697; latency to vocalize: 0.885; latency to move towards or away from the loudspeaker: 0.799; distance moved: −0.742; number of emitted calls: −0.904). Negative PC scores thus indicate a strong reaction, with shorter latencies, close approach to the speaker and calls in response to the playback. When we modified call pulse rate, males responded equally to the three experimental signals (figure 4*b*; Wilcoxon matched pairs tests on PC1 scores: *n*=10, Z=0.051, *p*=0.959 for 1.7 Hz versus 1 Hz; *Z*=0.357, *p*=0.721 for 1.7 Hz versus 3 Hz; *Z*=0.15, *p*=0.878 for 1 Hz versus 3 Hz; for PC2 scores *p*=0.241, *p*=0.203 and *p*=0.444, respectively). When we modified the number of pulses per call, we similarly observed no significant differential responses from the tested males, although there was a trend for males to respond more strongly when the number of pulses was higher (figure 4*c*; Wilcoxon matched pairs tests on PC1 scores: *n*=10, Z=0.42, *p*=0.674 for 14 versus 7 pulses; Z=1.78, *p*=0.074 for 14 versus 21 pulses; Z=1.68 *p*=0.09 for 7 versus 21 pulses; for PC2 scores *p*=0.401, *p*=0.074 and *p*=0.721, respectively). Males did not show differential responses to signals with modified spectral composition (figure 4*d*; Wilcoxon matched pairs tests, PC1 scores: *n*=10, *Z*=1.07, *p*=0.284; PC2 scores: *n*=10, *Z*=0.968, *p*=0.333).

### 3.4 Individual vocal signatures

**Figure 5.**
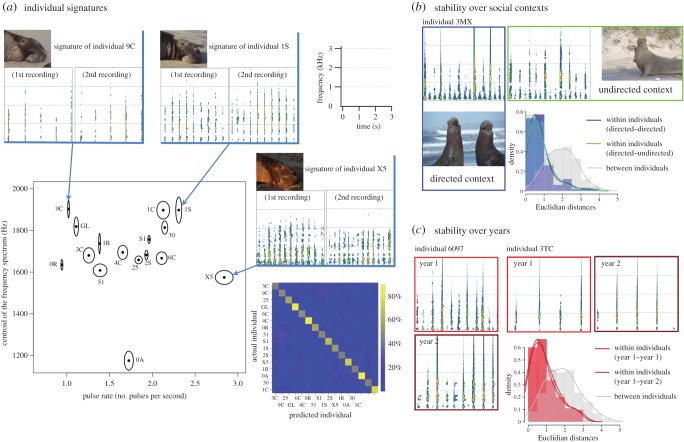
Individual signatures of the acoustic displays of male northern elephant seals. (*a*) As shown by the central graph and accompanying spectrograms on the sides, calls can be reliably assigned to individuals using two acoustic parameters (mean±s.e.): the centroid of the frequency spectrum and the number of pulses per call (the two main factors that separate individuals on the first discriminant function of the cross-validated DFA). The confusion matrix provided is obtained from the cross-validated DFA. It shows by colouring cell (*i*,*j*) the conditional probability of guessing that the test call came from individual *j* when in fact it was emitted by *i*. The yellow diagonal of the matrix underscores the high probability of correct classification (average=61.3% versus chance=6.3%, see text for details), highlighting the strength of the individual signatures. (*b*) Both spectrograms illustrate the consistency of an individual's calls in two different social contexts (calling alone and calling to a rival). The distribution of the Euclidian distances (density curves) underscores the similarity of calls within and between contexts (in the two-dimensional space defined by the calls' frequency centroid and the pulse rate; *n*=8 individuals, 2–6 calls individual^−1^, see Material and methods). (*c*) Both pairs of spectrograms illustrate the consistency of an individual's calls over successive years. The distribution of Euclidian distances (density curves) shows the remarkable proximity of calls within and between years (*n*=10 individuals, 5–6 calls individual^−1^ year^−1^). The calls represented by spectrograms in the figure are available as electronic supplementary material, audio S1.

Our qualitative observation that experienced observers could identify males solely by their calls was supported by a quantitative cross-validated and pDFA. The results of the cross-validation step showed that individual identification on the basis of six spectral and three temporal acoustic parameters was highly reliable (average rate of correct classification=61.3%, range: 35.9–99.5%; chance=6.3%; *n*=16 adult males with 15.8±3.5 calls/individual, range: 9–20; *p*<0.001; see classification matrix in figure 5*a*; electronic supplementary material, audio S1). The two main acoustic factors separating individuals on the first discriminant function were one temporal and one frequency parameter: call pulse rate and the centroid of the call frequency spectrum (table 4). The combination of these two cues was sufficient to characterize the unique acoustic space of each individual (figure 5*a*), even without further consideration of notable differences in fine-scale pulse structure.

**Table 4.** Summary of acoustic parameters measured. (Note that LD1 gives the loadings of acoustic parameters for the first discriminant function used to classify calls from different individuals.)

acoustic parameter	*n*	no. calls per male±s.d. (range)	group mean±s.d.	range of mean values for individuals	LD1
temporal					
duration (s)	16	15.8±3.5 (9–25)	10.3±3.7	5.7–19.5	0.192
pulse rate (Hz)	16	15.8±3.5 (9–25)	1.73±0.5	0.94–2.84	−0.795
no. pulses	16	15.8±3.5 (9–25)	15.6±7.5	7.8–35.7	−0.411
spectral					
*F*_max_ (Hz)	16	15.8±3.5 (9–25)	460±161	232–725	−0.067
centroid (Hz)	16	15.8±3.5 (9–25)	1700±175	1574–1902	0.656
Q25 (Hz)	16	15.8±3.5 (9–25)	643±127	263–781	0.064
Q50 (Hz)	16	15.8±3.5 (9–25)	1209±216	543–1473	−0.028
Q75 (Hz)	16	15.8±3.5 (9–25)	2367±296	1624–2774	−0.426
−12 dB bandwidth (Hz)	16	15.8±3.5 (9–25)	1077±347	241–1578	−0.074
amplitude					
dB_peak_ re: 20 μPa at 1 m	15	4	126.2±3.3	120.4–130.2	

By recording 10 individuals over consecutive years, we found that their vocal signatures were stable over at least two seasons (figure 5*c* and table 5). Further, comparisons of Euclidian distances for calls recorded for eight individuals within and between social contexts (undirected versus directed calls) showed that an individual's call exhibited the same signal structure and amplitude regardless of the social condition during which it was emitted (figure 5*b* and table 5).

**Table 5.** Comparison of individuals' calls between years and across social contexts.

	mean Euclidian distances^a^	
individual	within year	between years
X579	1.257	1.898
25C	0.932	0.934
GL	1.36	1.011
4C	1.25	0.986
6Z	0.668	0.799
9R	1.21	1.413
2BR	1.009	0.984
3TC	0.554	0.868
3MX	0.906	0.774
6097	0.502	0.791
comparison^b^	*p*>0.05	

	within directed context	between directed and undirected contexts
2BR	0.701	0.574
3MA	1.196	1.016
3MX	0.898	0.981
3TC	0.318	0.252
6LI	0.766	0.769
6RA	0.680	0.735
8IC	1.884	1.559
6097	0.651	0.545
comparison^b^	*p*>0.05	

^a^Euclidian distances, calculated in the two-dimensional space defined by calls' centroid of the frequency spectrum and pulse rate, were determined for the calls from males recorded on two successive years or on two different social contexts (e.g. calls directed and non-directed to a particular individual).

^b^There is no significant difference between the distance separating an individual's calls recorded in the same year and the distance between calls recorded in two consecutive years (*n*=10 males, Wilcoxon matched pairs test, *z*=−0.64, *p*>0.05). Similarly, there is no difference in calls emitted in the directed context, relative to those emitted in non-directed context (*n*=8 males, Wilcoxon matched pairs test, *W*=22, *p*>0.05).

### 3.5 The influence of social knowledge on the behavioural responses of males

**Figure 6.**
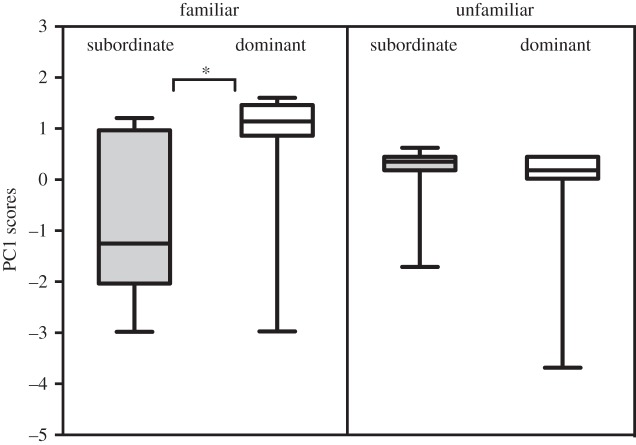
The conditional strategies of beta males. Panels show the behavioural responses exhibited by beta males when challenged with playbacks of lower ranking males (subordinate) or higher ranking males (dominant). The behavioural responses are expressed by composite scores (PC1 scores) that integrate the assessment of several behavioural parameters (see text for details): lower PC scores indicate a stronger aggressive reaction (shorter latencies, calls produced during the playback and an approach to the loudspeaker), while positive scores indicate a retreat. The target males showed strong differential movements towards or away from the speaker when the calls of familiar subordinate or dominant individuals were presented (left panel, Wilcoxon test, *n*=10, *z*=2.547, *p*=0.011). Conversely, the playback of calls from stranger individuals hardly elicited a behavioural reaction (right panel, Wilcoxon test, *n*=10, *z*=1.069 *p*=0.285). The whiskers on the box plots show min-max.

Beta males presented with calls from familiar dominant and subordinate rivals responded aggressively to the calls of their subordinate opponent by approaching the loudspeaker and vocalizing (i.e. negative PC scores), while they quickly moved away without calling (i.e. positive PC scores) upon hearing the calls of their dominant rival (Wilcoxon matched pairs test on PC1 scores, *n*=10, *Z*=2.1915, *p*=0.028; on PC2 scores: *n*=10, *Z*=1.68, *p*=0.09; figure 6; see the electronic supplementary material, video 2). The first two components of the PCA performed on behavioural measurements showed eigenvalues of more than 1, and explained 53% and 28% of the total variance, respectively. Distance moved, latency to vocalize and number of calls were correlated to PC1 (all positively except latency to vocalize), while latency to orient and latency to move were positively correlated to PC2. In a subsequent experiment, the same dominant–subordinate treatments were presented to 10 beta males of similar status from a distant colony. In this case, the focal males were unfamiliar with the callers. We observed no differential response to the calls of high-ranking and low-ranking strangers (Wilcoxon matched pairs test on PC1 scores, *n*=10, *Z*=0.652, *p*=0.515; on PC2 scores: *n*=10, *Z*=0.059, *p*=0.952; figure 6).

**Figure 7.**
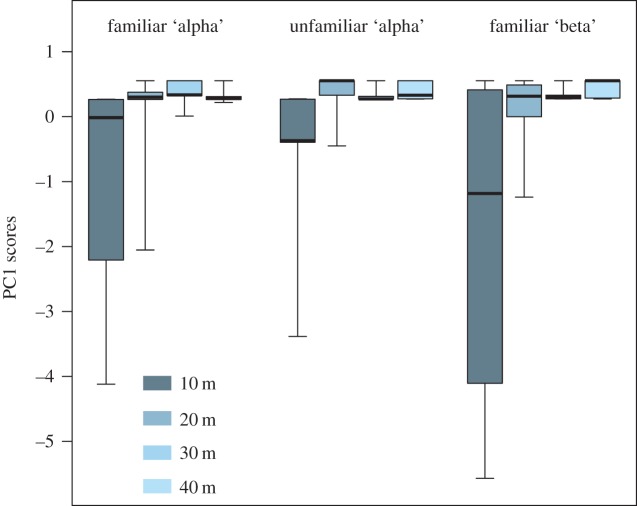
Behavioural response of alpha males to playbacks of calls from different male categories (familiar alpha, unfamiliar alpha, familiar beta) at different distances to illustrate the approach of an intrusive male. A negative PC score indicates a strong reaction (shorter latencies, calls produced during the playback and an approach to the speaker). Alpha males (*n*=5) showed a strong reaction only when the speaker was placed at 10 m (closest distance), regardless of familiarity (familiar/stranger) or social status (alpha/beta) of the caller. The whiskers on the box plots shown min-max.

Alpha males that were challenged with calls from nearby alpha males, familiar beta males and unfamiliar alpha males did not exhibit differential responses ($\chi_{2}^{2}=0.257$, *n*=5, *p*=0.879; figure 7). Only the distance of the playback significantly influenced their reactions to intruders' calls ($\chi_{1}^{2}=8.04$, *n*=5, *p*=0.005), with post-hoc comparisons indicating that behavioural responses were strongest (i.e. negative PC scores) when the loudspeaker was closest, at 10 m (Tukey tests, between 20 and 10 m: *p*=0.005, 30–20 m: *p*=0.748, 40–30 m: *p*=0.975; figure 7).

## 4. Discussion

Exploring the mechanisms that sustain dominance hierarchies in animal groups is necessary to understand the dynamics of social interactions in situations where individuals compete for resources. This study reveals that signals produced by male northern elephant seals during rival assessment do not necessarily convey information about the motivational state or resource-holding potential of individuals. Instead of using signals to assess aggressive intention or fighting ability, individuals may need only to learn to associate the outcomes of previous interactions with reliable cues emitted by competitors. This social knowledge based on individual recognition enables competitors to choose between alternative behavioural responses (e.g. attacking, retreating or observing), thereby increasing opportunities to gain access to desired resources under the least costly conditions. In the social system of the northern elephant seal, the few alpha-status males defend their harem from any intrusion, while less dominant beta males recognize their opponents and respond conditionally on the basis of prior experiences. Here, we discuss the selection pressures that favour such a communication system, which relies on individual signatures and associative learning. We also consider how competitors assess and manage information about their social network, and emphasize the importance of combining descriptive and experimental methods when attempting to understand signalling strategies.

Reproduction in northern elephant seals combines an extreme level of intra-sexual competition with an extended period of fasting, and therefore imposes enormous energetic costs on males [16]. Individuals must balance their time between vigorously competing for access to females while minimizing energy and water loss, as well as avoiding harm. Substituting fighting and chasing with calling may represent the most efficient strategy in this system. We have shown that the acoustic displays produced by males serve as identity signals—that is, signals requiring only that senders and receivers have prior knowledge of one another and that they remember the outcome of previous competitive interactions [37]. While this kind of recognition has been observed to sustain dominance relationships within stable, territorial groups [38], far fewer studies have evaluated the role of individual recognition in maintaining these relationships in more fluid, non-territorial social environments.

The finding that male northern elephant seals can rely on individual acoustic signatures to assess their opponents is surprising given the more ‘honest’ signalling systems of mammals that compete for access to females groups rather than territories (e.g. [5]). From a strictly correlational standpoint, while some acoustic features of northern elephant seal displays were associated with morphological traits, there was no predictive relationship between these call parameters and the social dominance of callers. The extent to which size and status are linked is less clear: while we found no correlation between morphological traits and dominance rating among adult males, another study reports a positive association [39]. Irrespective of the relationship between phenotype and dominance, this study provides direct experimental evidence that—while some call features encode morphological traits—males *do not* attend to the available honest information during competitive encounters. Thus, it appears that hierarchical structure among the elite males that survive to physical maturity may be more influenced by the dynamics of social interactions than the advantages associated with size or strength [40–42]. In this highly competitive system, cues associated with phenotype or motivation may not be informative enough for males to discriminate between the few top contenders. Signals conveying individual identity are apparently more informative, and a male's success appears to rely on his ability to effectively manage this social knowledge throughout the breeding season.

To function effectively, such a system based on associative learning requires fidelity to a given breeding location, stable individual vocal signature, and the ability to recognize and remember the vocal signatures of other males. Northern elephant seal males show strong breeding-site fidelity [11]. This allows males the opportunity for repeated interactions with individuals in a given social hierarchy, as shown in this study. Reliable vocal signatures encode the identity of callers, regardless of social context. This benefits male callers through ease of recognition by subordinate rivals who are likely to retreat from contests. Males also use vocalizations to recognize more dominant, familiar opponents, and thus avoid conflicts that they are likely to lose. Paired with associative learning capabilities that allow individuals to link acoustic signatures to social consequences, the use of identity signals may produce an evolutionarily stable strategy within the context of dominance interactions within relatively large, social groups [43]. Moreover, since individual vocal signatures remain stable over successive years, it is possible that males may recognize familiar rivals across breeding seasons. Prior to each season, the relative dominance status of individuals may thus be influenced by the long-term memory of past competitors. However, multi-year recognition of rivals warrants further investigation in this species.

We have found that male northern elephant seals are capable of accurately identifying familiar competitors on the basis of their calls, and show flexibility in their responses to threats depending on their dominance status and the familiarity of the caller. Beta males show a conditional behavioural strategy in response to challengers, responding appropriately to familiar males of known relative hierarchical status, while showing no specific reaction to the calls of strangers. We do not propose the males exposed to unfamiliar calls were indifferent, but rather, that lack of response may represent the safest strategy when assessing the calls of unfamiliar challengers. Mid-ranking males may have a good deal to gain by asserting dominance over new contestants, but a substantial amount to lose if their new opponent is far larger or more motivated to attack. This dichotomy in motivation to respond may leave mid-ranking males at a draw when first assessing the calls of unfamiliar opponents, and in these cases males may require additional information (including visual or seismic cues) about their opponent before deciding to attack or retreat. Once a male has reached alpha status and has gained a harem-controlling position, his motivation to defend against encroaching males is amplified. Alpha males appear to be highly responsive to the calls of any males, regardless of familiarity or social status, once they perceive an immediate threat. The behavioural plasticity of males highlights the importance of individual strategy in this system, and warrants further investigation in other breeding systems in which males compete for access to breeding rights.

In our study, male elephant seals were observed to engage with as many as 43 opponents over the course of a single breeding season. An individual's success within this relatively large and dynamic social system depends on managing knowledge of dominance relationships, regardless of whether this information is obtained through interaction or observation. As the amount of information stored by individuals may be limited, males could sort competitors into different relative social categories, and continuously update these categories throughout the breeding season. For example, a beta male could classify familiar competitors into a few functional categories, such as ‘alphas’, ‘higher ranking males’ and ‘lower ranking males’ [44,45], and respond similarly to the different individuals within each category. The level of accuracy and persistence of this kind of social knowledge remains to be investigated in this species.

## 5. Conclusion

The communication system of male northern elephant seals emphasizes the role that signalling plays during contests over resources, as well as conditional behavioural strategies for conflict resolution gained from social experience. An initial look at this system would suggest that the maintenance of hierarchical relationships relies on acoustic cues conveying resource-holding potential [5,46–48]. While we found positive correlations between body size and a few acoustic features, playback experiments showed that male northern elephant seals do not use these phenotype-linked cues to assess their rivals. Rather, listeners attend to and remember individual vocal signatures experienced during previous contests. These findings demonstrate the importance of using experimental approaches to confirm the biological function of animal acoustic signals. Several recent studies have used correlational approaches alone to argue that phenotypically ‘honest’ signals are used to settle conflicts between competitors (e.g. [7,30,31,46–48]). Given the growing interest in status signalling and selective pressures influencing the structure of animal social networks [49–51], we advocate that further studies combine both descriptive and experimental methods to establish a true understanding of the information gained by the exchange of specialized signals.

## Supplementary Material

Audio 1 (audio file).

## Data accessibility

Data files from this study can be found in the Dryad database (http://dx.doi.org/10.5061/dryad.6g06h).
